# Unveiling the flexural strength of corroded prestressed self compacting concrete beams enhanced with M-sand and polypropylene fibres

**DOI:** 10.1038/s41598-025-01264-z

**Published:** 2025-05-12

**Authors:** Yamuna Bhagwat, Gopinatha Nayak

**Affiliations:** Department of Civil Engineering, Manipal Institute of Technology, Manipal Academy of Higher Education, Manipal, Karnataka 576104 India

**Keywords:** Accelerated corrosion, Deflection, Flexural strength, M-sand, Prestressed concrete beam, Polypropylene fibre, Self compacted concrete, Ultimate load, Engineering, Civil engineering

## Abstract

Prestressing steel corrosion is one of the barriers to the serviceability of prestressed concrete structures. The presence of aggressive environmental conditions leads to a reduction in the efficiency of the structures by degradation. Hence, untimely deterioration of the structures before completion of the expected service life is of great concern for engineers and researchers. The development of corrosion is faster and more severe in prestressed steel than in normal steel because of the high stress in prestressing steel. Therefore, a detailed investigation of the prestressed concrete structure under a corrosive environment is essential. This particular study focused on studying the corrosion effect on flexural behaviour of prestressed self compacting concrete beams made of M40 and M60 grade mixes using M-sand as fine aggregate, also with and without polypropylene fibre. The beam specimens were artificially corroded by the accelerated corrosion method, and the flexural strength of the corroded and non corroded prestressed concrete beam specimens were studied under four point bending method. The comparison study of prestressed concrete beams with and without polypropylene fibre showed that corrosion levels obtained in the corroded prestressed concrete beam specimens with fibre were less than the corroded prestressed concrete beam specimens without fibre at a constant period with a constant current. The corrosion levels obtained in M60 self compacting concrete were less than that of M40 self compacting concrete specimens. Also, corrosion of the strand reduced the cracking load, ultimate load, ultimate deflection, energy absorption capacity and stiffness of prestressed concrete beam specimens. The study concludes that the addition of polypropylene fibre to the self compacting concrete mixes improves the corrosion resistance of prestressed concrete beam and the flexural performance of the corroded prestressed concrete beam.

## Introduction

Prestressed concrete structures are normally reinforced with high strength steels. Bond behavior between concrete and steel is considered to be a key role in the performance of prestressed concrete structures. In the process of prestressing the concrete, the tensile force in the steel must be transferred to the concrete as compression through the bond between steel and concrete. Thus, prestressing steel has to experience very high level tensile stresses to counteract the external loads on structural elements. Therefore, stresses in the steel and the corrosive environment together increase the possibility of stress corrosion cracking. The spreading of the corrosion reduces the tensile strength of steel and bond strength^1,2^. Therefore, in prestressed concrete structures, the severity of possible repercussions of steel corrosion is greater than that of reinforced concrete structures. The failure of the corroded prestressing steel specimens depends on the type of corrosion, type of loading (i.e., cyclic load, dynamic load, static load) that the steel experiences and steel properties. The critical pits formed on steel due to corrosion reduce the sectional area and sectional stiffness of steel^3^. Further, the reduction in the sectional area of steel reduces the elongation of the steel due to localized corrosion pit formation^4,5^. The studies showed that the prestressing steel corrosion significantly reduces the flexural capacity of the beam and beam failure changes from ductile failure to brittle failure^6,7^. Also, with the increase in the corrosion level, the cracking load, yield load and ultimate load reduce significantly^8^. The defects in the steel concrete interface accelerate the corrosion initiation in steel bars^9^. In addition, with the increase in tension strain in the prestressing steel, the corrosion rate also increases^10^.

The degradation of reinforced concrete structural elements by corrosion is a universal problem that affects the performance of the structure. This is due to aggressive agents like deicing salt, chloride-contaminated aggregates and chloride ions from marine environments. The corrosion products form in the corrosion process of reinforcements cause expansive pressure on the surrounding concrete, which leads to spalling off the concrete and reduces the serviceability of the structure. However, the use of concrete mix with well dispersed fibres in the reinforced concrete beam help to retain the load bearing capacity. The optimum amount of fibres in concrete can increase its durability by reducing concrete erosion in inimical conditions. Also, these fibres restrains the cracks in the concrete and contributes to increase the durability of concrete^11–14^.

## Background study

The performance of the concrete is also one of the main concerns to improve the durability of the concrete structure. Since Self Compacting Concrete (SCC) has become very popular in the construction industry, SCC is used in this particular study. The experimental work done by Kumar et al.^15^ showed that in situ concrete properties are improved in SCC than in conventional compacted concrete. Also, they have found that fly ash based SCC performs better than ordinary portland cement beyond 90 days with improved in situ durability without altering strength properties. Arezoumandi et al.^16^ proved that the SCC shows better bond strength than conventional concrete. Also, the study^17^ showed that probability of rebar corrosion occurrence is lower in SCC mix compared to conventional concrete. The study by Soylev and Francois^18^ proved that the use of SCC gives better protection against corrosion because of the formation of a less porous and more homogeneous interface, since steel concrete interface quality influences the corrosion process. In addition, it is found that the use of polypropylene fibres (PPF) improves the durability of the concrete. Sun and Xu^19^ investigated the mechanism and reinforcing effect of PPF on the properties of concrete. Their results showed that PPF decreased the microvoids, and reduced the orientation and crystallization of Ca(OH)_2_. Also, compressive strength, flexural strength, bonding strength, dynamic and fatigue life performance were found to be improved for 0.9 kg/m^3^ of PPF content, and water penetration and mass loss were reduced.

The study by Karahan and Atis^20^ concluded that the combination of PPF and fly ash in concrete not much improved the compressive strength and elastic modulus but decreased the drying shrinkage and slightly improved the freeze-thaw resistance. Additionally, Gencel et al.^21^ investigated the fresh and mechanical properties of SCC concrete with fly ash and polypropylene monofilament fibres (fibre contents of 3, 6, 9 and 12 kg/m^3^). It was found that uniform distribution of fibre enhanced the strength of SCC by not much affecting workability. Furthermore, addition of PPF reduced the mass loss in the concrete specimen and increased the durability^22,23^. Also, presence of fibre improved the coefficient of chloride diffusion, lowered the coefficient related to free chloride and delayed the corrosion in reinforced beam^24^. Ramezanionpour et al.^25^ investigated the PPF effect on mechanical, physical and durability characteristics of the concrete. They found that the optimum amount of fibre increased the durability of concrete by reducing chloride diffusion, sorptivity and water penetration. The presence of fibres also increased the compressive and flexural performance of the concrete. Also, Lee et al.^26^ determined the flexural capacity of the normal concrete using high strength macro PPF of volume fraction 0.25, 0.5,0.75 and 1% for 30, 40, 60 MPa concrete. The results showed that more than 0.5% volume fraction improved the flexural capacity of the concrete beam. Similarly, Rajesh Kumar et al.^27^ studied the structural performance of the fibre reinforced SCC beam with corroded reinforcements. In this, glass chopped fibre and PPFs were used with SCC and determined the fresh and hardened properties of the concrete. Even though the corrosion of reinforcements reduces the structural performance, the study showed that the use of fibres elevates the deteriorated beam performances in terms of stiffness, flexural capacity and ductility.

In additon to the detailed study on concrete, a study on prestressing steel is also necessary to understand the corrosion mechanism and effect of corrosion on prestressed concrete structural elements. Since prestressing steel is subjected to a high stress, the stress levels found as a main factor for stress corrosion cracking^28^. The stress corrosion cracking develops “micro-cracking and micro-void” in the highly loaded steel because of the stress concentration in pits formed due to corrosion. It is proved from the experimental study conducted by Lee et al.^29^ that there is a linear relationship between prestressing load and corrosion rate. Similarly, Wang et al.^30^ investigated the corrosion morphology and mechanical behavior of corroded prestressing strands. They found that an increase in corrosion loss with an increase in stress level. Also, high stress level increased the spread of microcracks on the strand surface and they affected the ultimate strength of strand. Furthermore, an investigation done by Yoo et al.^31^ on naturally corroded prestressing steel strands extracted from two in-service bridges showed that the linear trend between measured sectional loss and corrosion depth. In addition, Diaz et al.^32^ explored the electrochemical behavior of high-strength steel wire when exposed to highly alkaline solutions and concluded that the presence of chlorides, oxygen availability, and temperature variations are the significant contributors to the initiation of corrosion in prestressing steel. A study by Shanglin Lv et al.^33^ concluded that stress in the strand promotes the corrosion of high strength steel, and also stress corrosion depends on the steel composition and microstructure. Li et al.^34^ showed from their study that prestressing force in a strand reduces with corrosion and corrosion deteriorates the tensile properties of the steel strand.

Wang et al.^35^ proved in their study that an increase in prestress in steel increases the cracking process due to corrosion and increases the rate of crack propagation in the prestressed concrete and correspondingly decreased the critical time for cover cracking. Also, Belletti et al.^36^ experimentally investigated the shear capacity of the naturally corroded prestressed concrete (PSC) beam and found that prestressing steel corrosion reduces the shear capacity of beam. Similarly, Jeon and Shim^37^ studied the flexural behaviour of post tensioned concrete beam with a corroded strand and found that the beam having strand corrosion at the mid span of the beam significantly reduced the flexural strength of beam. Jeon et al.^38^ experimentally studied the PSC beam with corrosion. The strand used in beam was corroded using impressed current technique and flexural strength was studied. Corrosion location, corrosion degree and number of corroded strands were considered as parameters. The result showed that the centrally corroded PSC beam shows more reduction in the strength than any other location. The degree of corrosion found to be proportional to the strength reduction of beam. With this, strand corrosion reduces the flexural capacity of the beam and increases the risk of brittle failure under service conditions^39^. Also, corrosion deteriorates cracking load, yield load, ultimate load capacity, flexural stiffness and deflection of beam with the corrosion degree^8,40^, beam failure mode changes to brittle in 8–11% corrosion level^41^.

**Table 1:** The chemical properties of PPC.

Element	Content in%
Silica (SiO_2_)	32.8
Iron oxide ($\hbox {Fe}_2\hbox {O}_3$)	5.7
Alumina	9.9
Calcium oxide	42.6
Sulphur trioxide	2.6
Magnesium oxide	1.5
Sodium oxide	1.2
Potassium oxide	0.4

**Table 2:** M40^42^ and M60 SCC mixes details used in study.

Mix contents	Mix details for M40	Mix details for M60
PPC content (kg/m^3^)	650	650
Water (kg/m^3^)	201.5	185
Fine aggregate (kg/m^3^)	741	741
Coarse aggregate (kg/m^3^)	738.09	738.09
Admixture dosage (%)	0.7	0.7
water/cement ratio (%)	0.31	0.285

**Table 3:** The performance of M40^42^ and M60 SCC with and without fibre.

SI. no.	Parameters	Average values			
		M40 SCC	M40 SCC with 0.15% PPF	M60 SCC	M60 SCC with 0.15% PPF
1	Slump flow results	730 mm	650 mm	670 mm	562 mm
2	T500 results	2 Seconds	2.5 Seconds	3 Seconds	3.5 Seconds
3	L-box results	0.91	0.86	0.905	0.89
4	V funnel results	7.6 Seconds	13 Seconds	11.3 Seconds	18 Seconds
5	Compressive strength (28 days)	65.03 N/mm^2^	68.57 N/mm^2^	69.3 N/mm^2^	71.7 N/mm^2^
6	Modulus of Elasticity	38.3 GPa	38.5 N/mm^2^	38.6 GPa	38.5 GPa
7	Spit Tensile strength	3.21 MPa	4.38 MPa	3.5 MPa	4.4 MPa
8	Flexural strength	4.27 MPa	4.4 MPa	4.42 MPa	4.56 MPa
9	Water absorption	0.42%	0.30%	0.45%	0.43%
10	Water penetration depth	11 mm	8 mm	7 mm	6 mm
11	Change in weight of 100mm cubes (kept in NaCl solution for 56 days)	3 gm	4 gm	3 gm	2 gm
12	Change in weight of 100mm cubes (kept in $\hbox {H}_2\hbox {SO}_4$ solution for 56 days)	38 gm	33 gm	32 gm	30 gm
13	Average time taken for crack initiation in corrosion study	501.5 hrs	522 hrs	505.6 hrs	524.25 hrs
14	RCPT test results	227.16 Coulombs	227.16 Coulombs	232.56 Coulombs	244.86 Coulombs

Prestressed concrete structures have taken the forefront in recent years due to innovations in the construction industry. However, corrosion is one of the barriers to the serviceability of the prestressed concrete structures. Therefore, a detailed investigation of the prestressed concrete structure under a corrosive environment is essential. This study necessitates a thorough investigation of the mechanical and durability properties of the structural elements and an effort to enhance their corrosion resisting capacity. Therefore, the present study attempts to understand the corrosion effect on the flexural behavior of prestressed self compacting concrete beams with and without PPFs. Also, the scarcity of natural sand makes the construction industry to lean towards using M-sand as fine aggregates. Thus, as a part of this study, prestressed self compacting concrete beams made of M-sand with and without PPF are cast, artificially corroded and analysed under four point bending and results are finally compared with the noncorroded prestressed SCC beam. Our experimental study consists of 12 PSC beams with SCC. Out of these, four are PSC beams without PPF and four are PSC beams with PPF, which are corroded by the accelerated corrosion method. The other four PSC beams without corrosion are considered as reference beams.

## Materials and methodology

### Concrete mix

In this study, Portland Pozzolana Cement (PPC) is used as binding material, M-sand as fine aggregates, 12 mm size crushed angular aggregates as coarse aggregate.

The PPC (Confirming to IS 1489-2015^43^) with 32% fly ash content and 2.92 as specific gravity is used for the study and its chemical composition is tabulated in the Table 1. And the PPFs used having diameter of 24 microns, length of 12 mm and an aspect ratio of 500 with specific gravity 0.9. Also, potable water and polycarboxylate ether based superplasticizer is used in the preparation of concrete mix. After many trials following EFNARC 2005 and IS 10262-2019^44,45^ guidelines SCC mix for M40 and M60 grade are finalised. The details of the SCC mix used for the study is tabulated in Table 2. After many trials trying for different percentage of PPF content, 0.15% volume fraction of PPF content in concrete is considered as optimum value. Then, performance of the concrete mixes are studied with and without PPF fibre for its fresh, hardened and durability properties, and the result obtained are tabulated in Table 3. Results showed that, addition of PPF lowers the workability of the SCC considerably. But the optimum percentage of PPF content improved the mechanical properties and durability with good workability. Addition of optimum PPF content reduced the water ingression in concrete by increasing the resistance of concrete to the external agents also enhanced the crack initiation time in corrosion study. After a detailed study of SCC M40 and M60 mixes with and without PPF, these concrete mixes are used for the preparation of PSC beams.

### Steel strand

The prestressing steel of 7-ply strand with diameter 12.7 mm, cross sectional area 98.7 mm^2^ and characteristic strength 1860 MPa is considered for study. Also, it is having minimum breaking strength of 183.7 kN and nominal weight of 0.775 kg/m.

### Types of specimen considered for study

The type of specimens considered are as follows : two numbers of corroded PSC beams, two numbers of corroded PSC beams with 0.15% fibre and two numbers of reference beams (which are not corroded) are examined for both M40 and M60 grade SCC, totaling six specimens for each grade.

### Specimen geometry and reinforcement

**Fig. 1:**
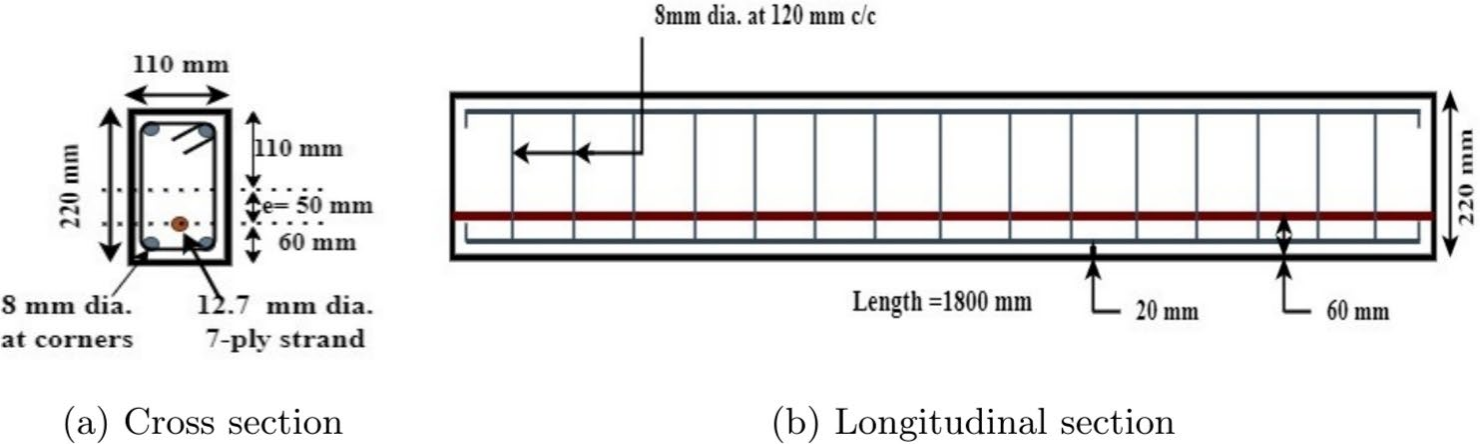
PSC beam details.

All the PSC beam specimens considered for study are having identical cross sectional areas of 110 × 220 mm and a span of 1800 mm. The details of the beam specimen are shown in Fig. 1. The beam specimens are prestressed with a single strand, placed at 50 mm eccentricity, at the middle bottom portion of the beam keeping 60 mm cover with initial prestressing force of 41 kN. Beam specimens are also reinforced with 8 mm diameter non prestressed steel four in numbers placed each at all four corners of the beam with a nominal cover of 20 mm. Shear reinforcement consists of 415 grade HYSD steel having 8 mm diameter and placed at 120 mm c/c. Due to the instrumental limitation, we adopted this particular dimension and initial prestressing force for the study.

### Casting and curing of PSC beam specimens

**Fig. 2:**
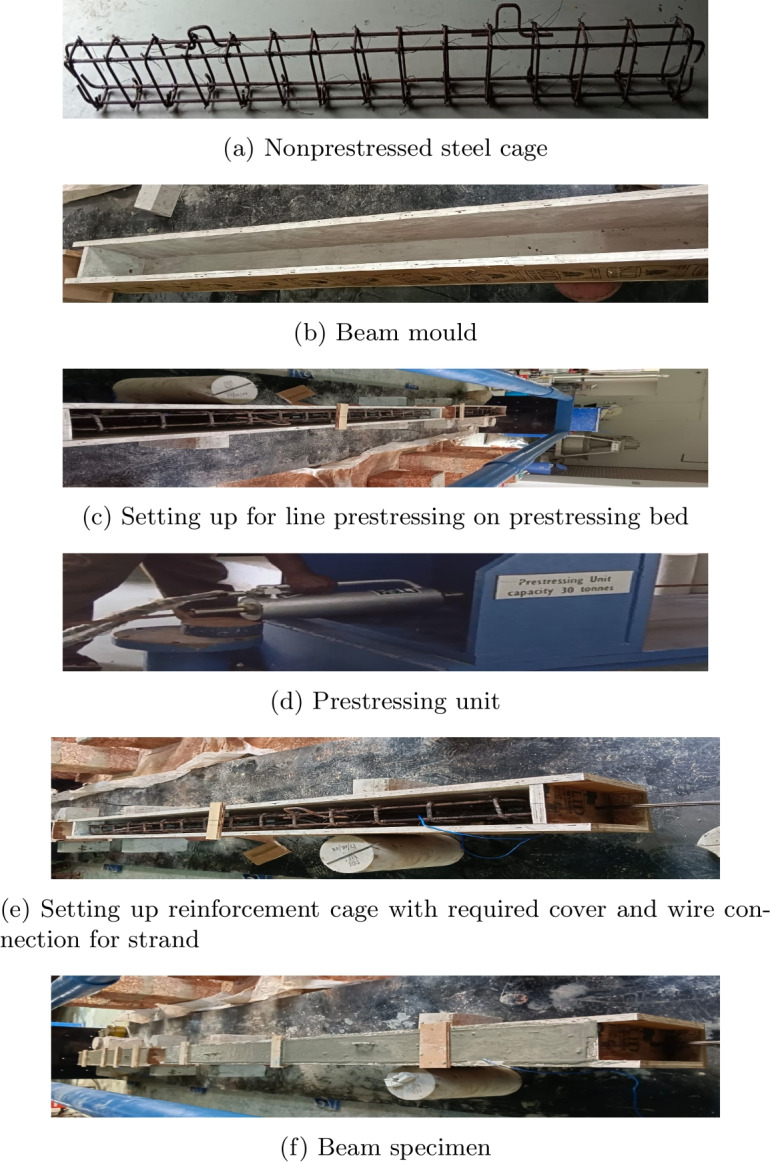
Casting of PSC beam specimens.

**Fig. 3:**
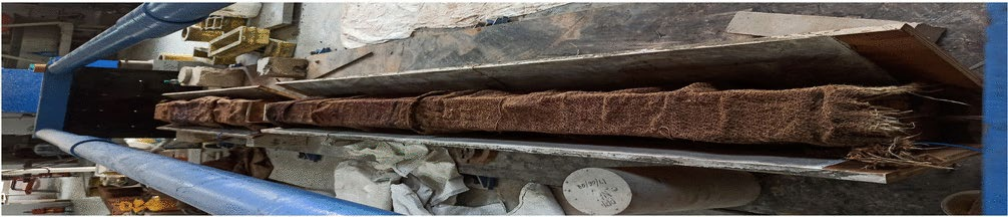
Curing of beam specimens.

There are different methods of casting prestressed beam specimens. In this study the line method of casting the PSC beam is adopted. Since beam specimens adopted have small lengths, prestressing is done only from one side. Figure 2 represents the line method of casing PSC beam and the method followed. Using prestressing bed of length 6000 mm, two number of beams of span 1800 mm are cast at once. After casting of beam specimens, the curing is done for 28 days by watering it. The curing of the beam specimen is shown in Fig. 3. The prestress is transferred to the concrete once it attains 28 days of strength which it is designed for. Then, the beam surface is observed for cracks and it is shifted to an accelerated corrosion tank for the corrosion process.

### Accelerated corrosion of PSC beam specimens

**Fig. 4:**
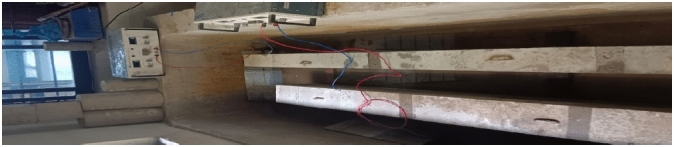
Accelerated corrosion of PSC beam.

After 28 days of curing, the prestress is transferred to the concrete beam specimens after ensuring the achievement of the required strength of concrete by cube testing, which is cast at the time of beam casting. Later, beams are kept in an accelerated corrosion tank which has a 5% NaCl solution for corrosion. The impressed current technique is used to incorporate the corrosion to the strand specimen. The current is setup only after 24 hrs of keeping the beam in electrolyte, by allowing electrolyte to get saturated. Corrosion setup is done by connecting the strand in the beam to the anode and stainless steel plate to the cathode of the DC power supply as shown in Fig. 4, impressed the current of 0.55 Amp for a period of 193 hrs. The corrosion period of 193 hrs is calculated using Faraday’s equation by considering maximum 6.5% corrosion percentage. There are many studies have been conducted using accelerated corrosion methods to incorporate corrosion. Nguyen and Lambert recommended the current density of 1000 µA/cm^2^ to study the reinforcement embedded in concrete^46^. After corrosion, nondestructive tests are conducted on beam specimens.

**Fig. 5:**
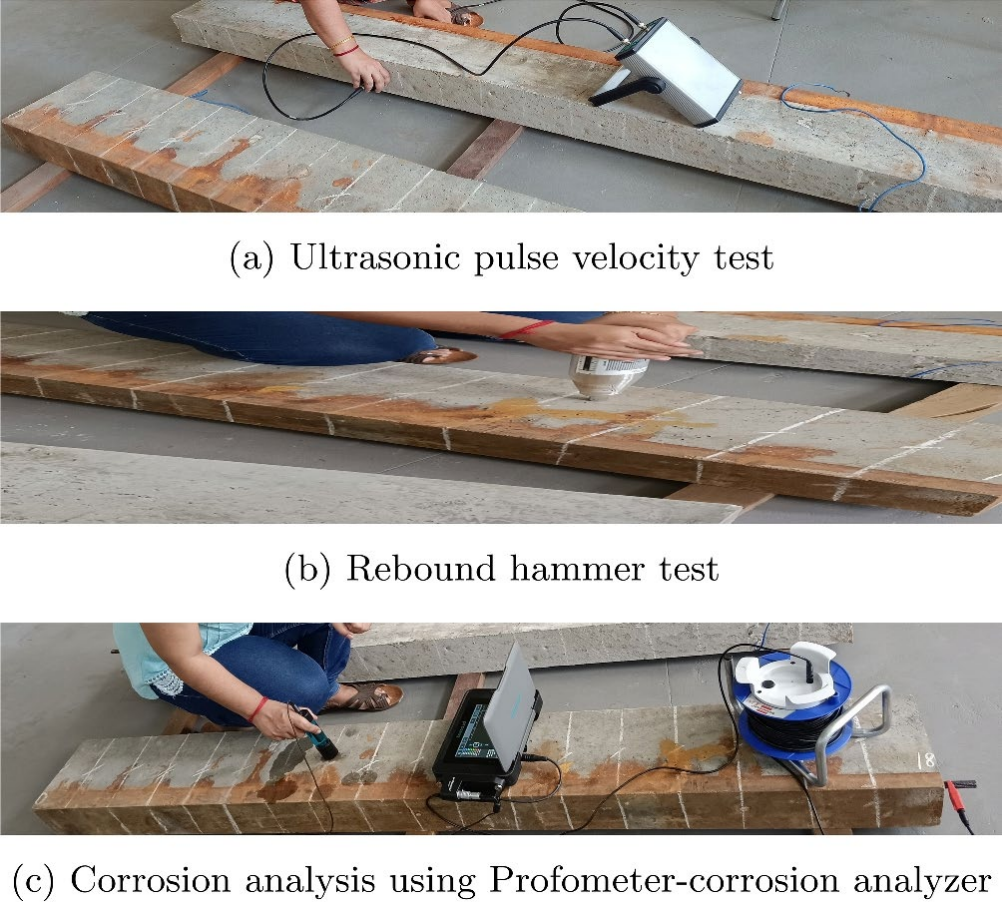
Nondestructive test on PSC beam specimens considered for study.

### Non destructive tests on PSC beam after accelerated corrosion

After accelerated corrosion, all the specimen’s surfaces are investigated for corrosion cracks and then to study the strength deterioration of beams Ultrasonic Pulse Velocity Test (UPVT) and rebound hammer tests are conducted. Later, a Profometer is used to understand the concrete contamination after keeping beams in 5% NaCl electrolyte for corrosion. Figure 5 shows the images of nondestructive tests on PSC beams.

**Fig. 6:**
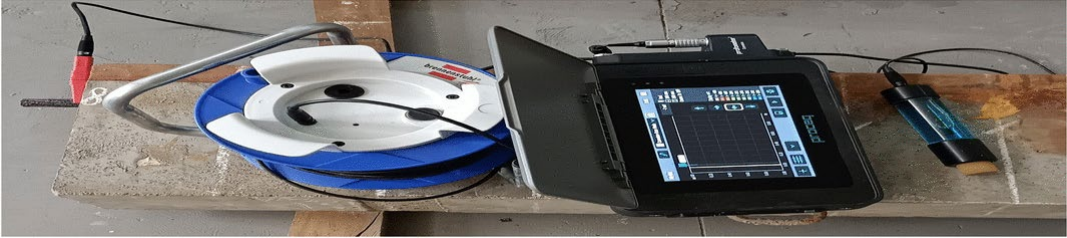
Profometer-corrosion setup.

#### Ultrasonic pulse velocity test (UPVT)

UPVT is conducted to assess the quality of concrete as per IS 13311 (Part 1)-1992^47^. The method consists of measuring the time taken to travel ultrasonic pulse velocity through the concrete by using following formula.

$$\begin{aligned} \text {Pulse velocity (m/s)} = \frac{\text {Path length}}{ \text {Travel time}} \end{aligned}$$

The Higher the pulse velocity, the higher the concrete quality in terms of uniformity and homogeneity of concrete. The direct method of measuring the pulse velocity is used for the study as shown in Fig. 5a.

#### Rebound hammer test

The rebound hammer test is the non destructive method of measuring the compressive strength of concrete. When the plunger of the rebound hammer pressed against the concrete surface, the spring controlled mass which it consists of rebounds. The test image is shown in the Fig. 5b. This rebound value is noted from the graduated scale on the rebound hammer. The amount of rebound value depends on the surface hardness. The higher the rebound number, higher the concrete strength. The concrete compressive strength is calculated from the graph given on the rebound hammer for the corresponding rebound number.

**Fig. 7:**
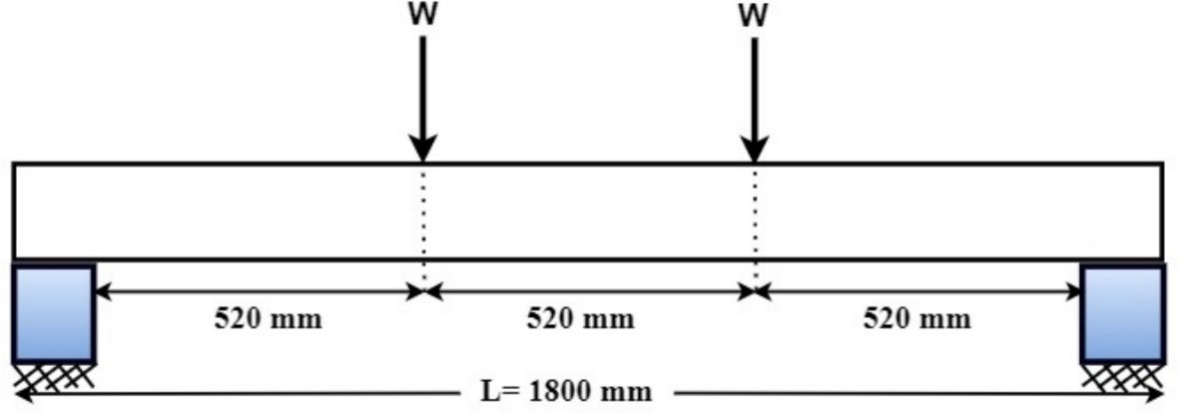
Diagrammatic representation of four point bending test setup.

**Fig. 8:**
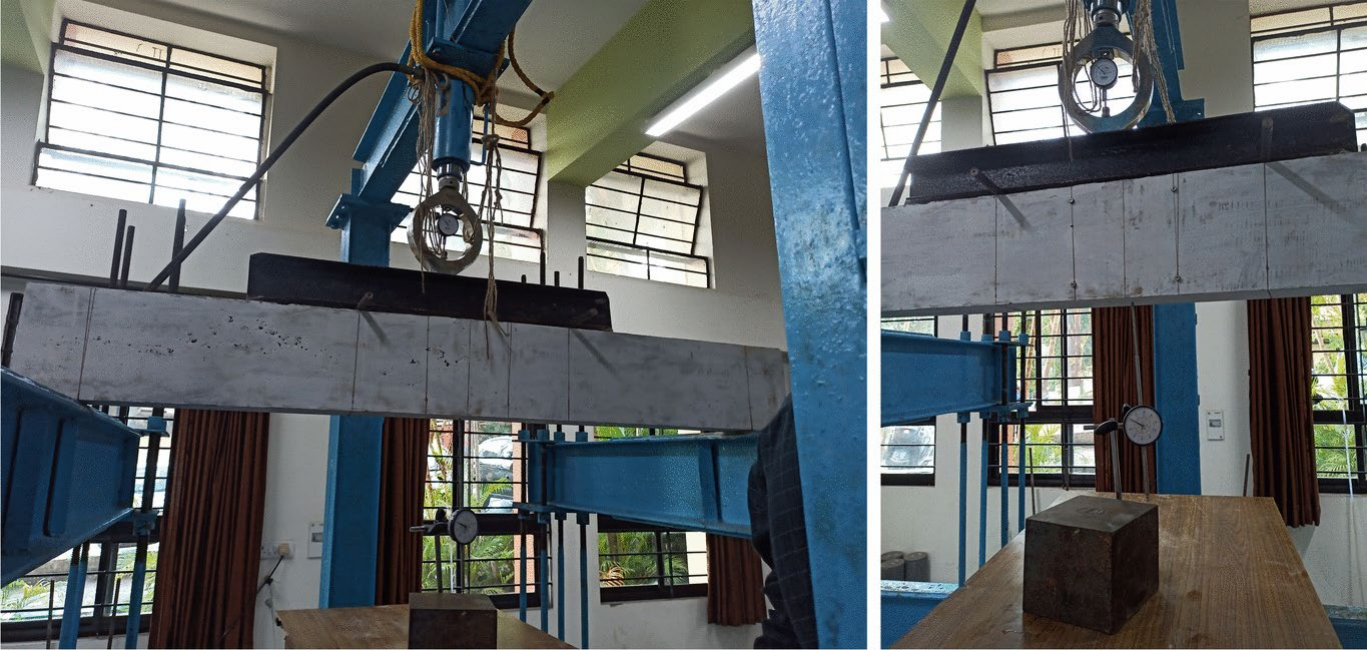
Flexural strength testing setup.

#### Corrosion analysis by profometer-corrosion

The profometer-corrosion is the advanced method of identifying the possibility of active corrosion of rebars by electrochemical properties of reinforced concrete. It is the qualitative method of measurement. It works on the principle of the half-cell potential technique. This instrument consists of a high-impedance voltmeter with a digital display and a copper rod electrode which is immersed in copper sulphate solution. The setup of profometer-corrosion is shown in Fig. 6. The spotting of highly contaminated concrete which influences corrosion initiation involves the measuring of negative half cell potential values. The copper rod electrode is the reference electrode and is moved in a grid over concrete surfaces to measure the potential. The 100 × 110 mm grid are marked on the beam. One side of the voltmeter is connected to the copper sulphate half cell rod which is held against the concrete surface and the other side is connected to a steel strand embedded in concrete shown in Fig. 5c. The potential difference between copper rod and steel strand is measured. The location with the most negative potential is the most contaminated concrete and is considered to have the highest possibility of localized steel corrosion.

### Flexural strength test on PSC beam specimens

The PSC beam specimens are tested under four point bending, schematically as shown in Fig. 7 and test images are shown in Fig. 8. The loading span is 1560 mm, and the load spacing is 520 mm. The dial gauge having 0.01 mm least count with 50 mm travel is set up to measure the vertical displacements. The loading frame with a capacity of 500 kN is used for the study. The test is conducted under a load control method with a loading rate of 5 kN/s. And for every satge of loading, the load and corresponding deflections are recorded. The cracking load, ultimate load and ultimate deflections are also noted. Later, the energy absorbed and stiffness of the beam specimens are calculated from the Load-deflection graph.

After conducting a flexure test on beam specimens, the steel strand is extracted by beam destruction. Then, the steel strand is cleaned and mass loss of the strand is recorded for calculating the corrosion degree.

## Results and discussion

All the test results conducted on the corroded and noncorroded PSC beam specimens are discussed below. Under each type of specimen, two specimens are cast and the average values of the test results are considered for the comparison study. The concrete cubes cast at the time of PSC beam casting are tested under compressive strength to know the characteristic strength of the mix used. Each PSC beams are cast individually due to the availability of only small concrete mixer, and cubes were also cast to check the compressive strength. Table 4 shows the compressive strength of those cubes cast for all the types of specimens considered.

**Table 4:** The 28 days compressive strength of the concrete mix used for casting PSC beam specimens.

Specimen type	Average compressive
	Strength (N/mm^2^)
Reference PSC beam of M40 SCC	46.5
Corroded PSC beam of M40 SCC	43.5
Corroded PSC beam with fibre of M40 SCC	43
Reference PSC beam of M60 SCC	64
Corroded PSC beam of M60 SCC	63
Corroded PSC beam with fibre of M60 SCC	64

### Concrete surface investigation after accelerated corrosion

After the accelerated corrosion, PSC beams are investigated for cracking due to the corrosion process. However, there are no cracks due to corrosion are observed. The study done by Rengaraju S et al.^48^ concluded that there is no visible stains or cracks or deflection are observed till 6% of the strand cross section is corroded. This is due to the deposition of corrosion products in interstitial space between the seven wires of the strand, which will not reach the concrete surface.

**Table 5:** The average values of UPVT and rebound hammer test results after accelerated corrosion of PSC beam specimens.

Specimen type	Average	Average compressive	
	UPVT	Strength (N/mm^2^)	
	Result (m/s)	Top	Bottom
Reference PSC beam of M40 SCC	4263	41	43
Corroded PSC beam of M40 SCC	3934.5	37	38
Corroded PSC beam with fibre of M40 SCC	3896	38.5	37.5
Reference PSC beam of M60 SCC	4617.5	62	59.5
Corroded PSC beam of M60 SCC	4475	56.5	57.5
Corroded PSC beam with fibre of M60 SCC	4511	56	58.5

### Non destructive test results on corroded and non corroded specimens

#### UPVT

The UPVT is used for the assessment of concrete quality. It provides information on that whether the concrete has cracks, defects and uniformity, since pulse velocity depends on the density and elastic modulus of concrete, which in turn defines concrete quality relatively. The best results in UPVT are obtained by using the direct transmission method i.e. by placing the transmitting and receiving transducers on the opposite face of the concrete member. Because when ultrasonic pulse strikes the concrete surface the maximum energy is propagated at right angles to the face of the transmitting transducer and best results are obtained^47^. Therefore, the results obtained from the UPVT are tabulated in Table 5. As per the values given in IS 13311-1992^47^, the concrete quality is good for the reference beam and also for the corroded PSC beams even after keeping in 5% NaCl solution for 10 days.

#### Rebound hammer test

The rebound hammer test estimates the compressive strength of concrete. However, the result of this test depends on the surface evenness, presence of cracks and voids in the concrete, age of the concrete and presence of reinforcement. Also it is evident that result accuracy depends on the calibration of instrument^49,50^. An average of 15 rebound values are considered and converted as compressive strength by referring to the graph given on the rebound hammer. Table 5 presents the compressive strength from the rebound hammer test for the considered PSC beam specimens at the side face top and bottom location. The slight variation in compressive strength is observed from the result; however rebound hammer test underestimates the compressive strength values when compared to the compressive strength obtained by cube crushing in compressive machine^51^.

**Fig. 9:**
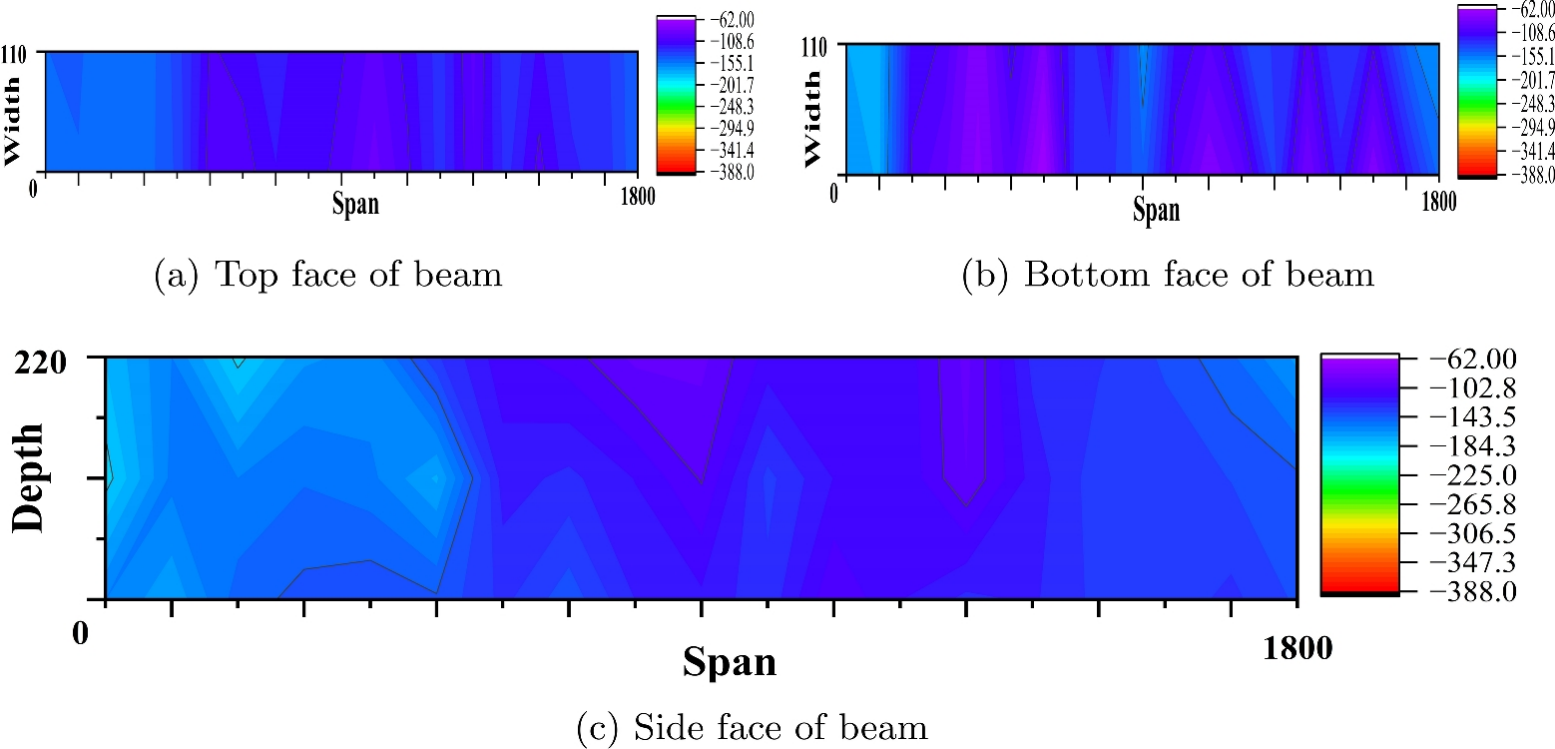
Equipotential plot of reference PSC beam made of M40 SCC (Beam dimension is in mm and potential values in mV).

**Fig. 10:**
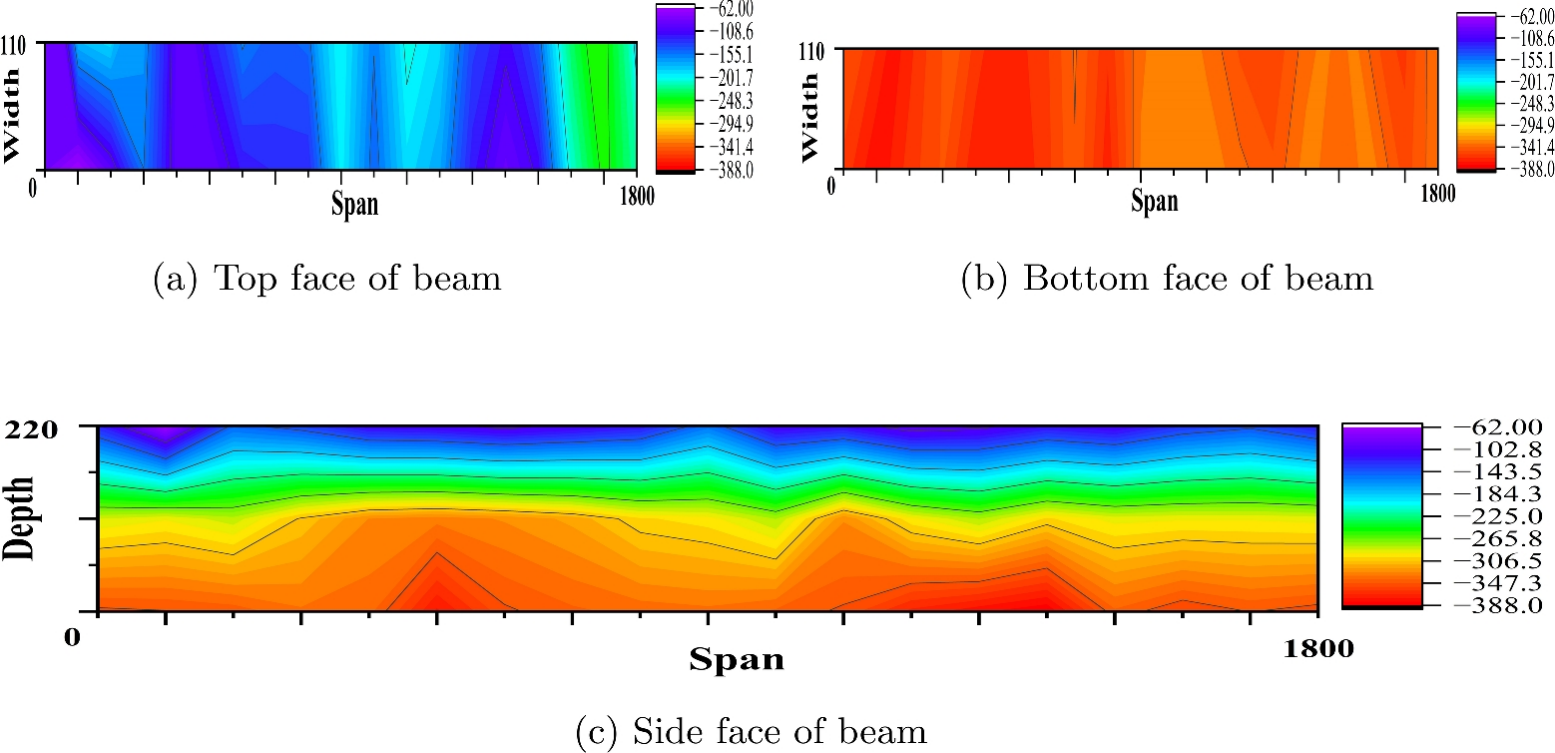
Equipotential plot of corroded PSC beam made of M40 SCC (Beam dimension is in mm and potential values in mV).

**Fig. 11:**
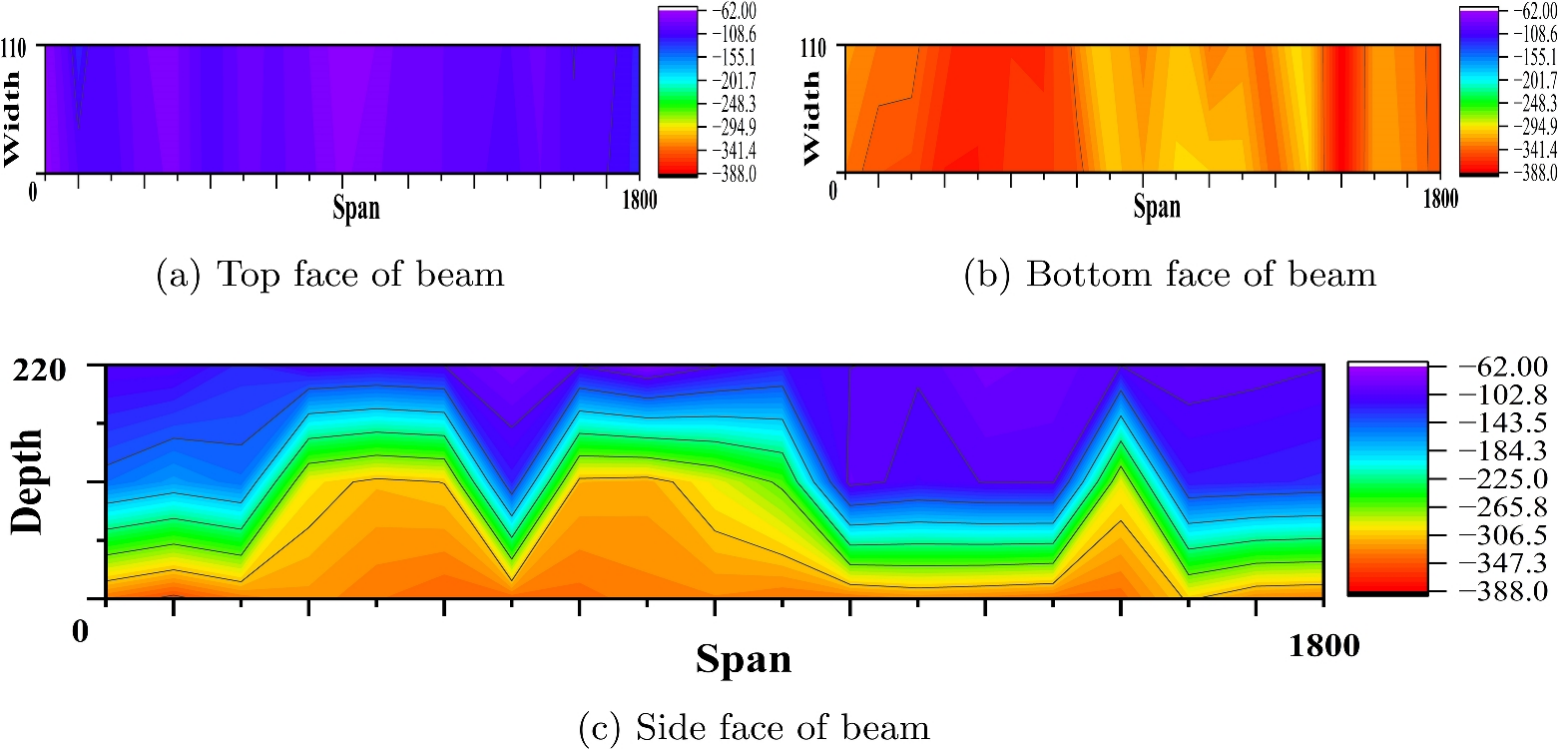
Equipotential plot of corroded PSC beam made of M40 SCC with 0.15% PPF (Beam dimension is in mm and potential values in mV).

**Fig. 12:**
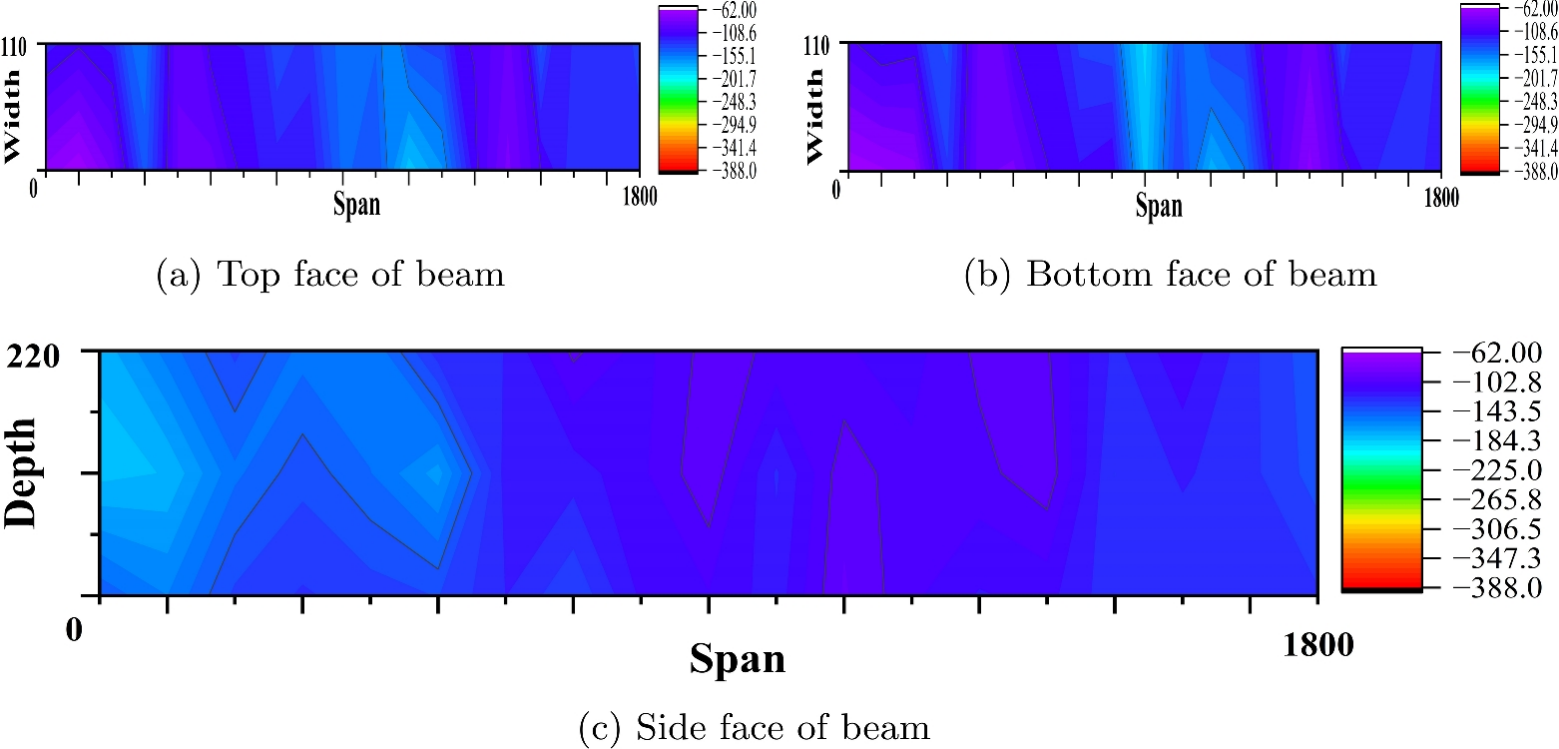
Equipotential plot of reference PSC beam made of M60 SCC (Beam dimension is in mm and potential values in mV).

**Fig. 13:**
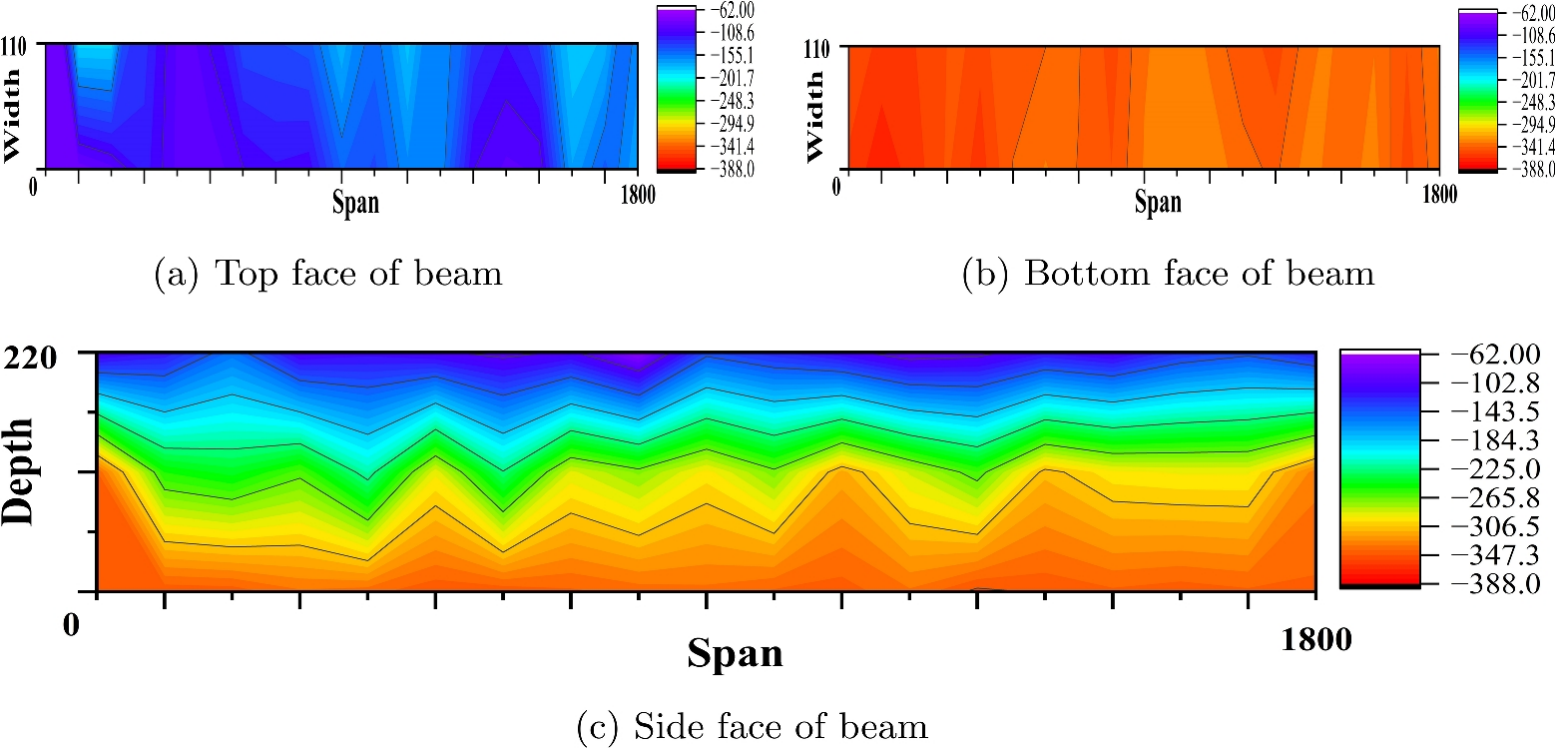
Equipotential plot of corroded PSC beam made of M60 SCC (Beam dimension is in mm and potential values in mV).

**Fig. 14:**
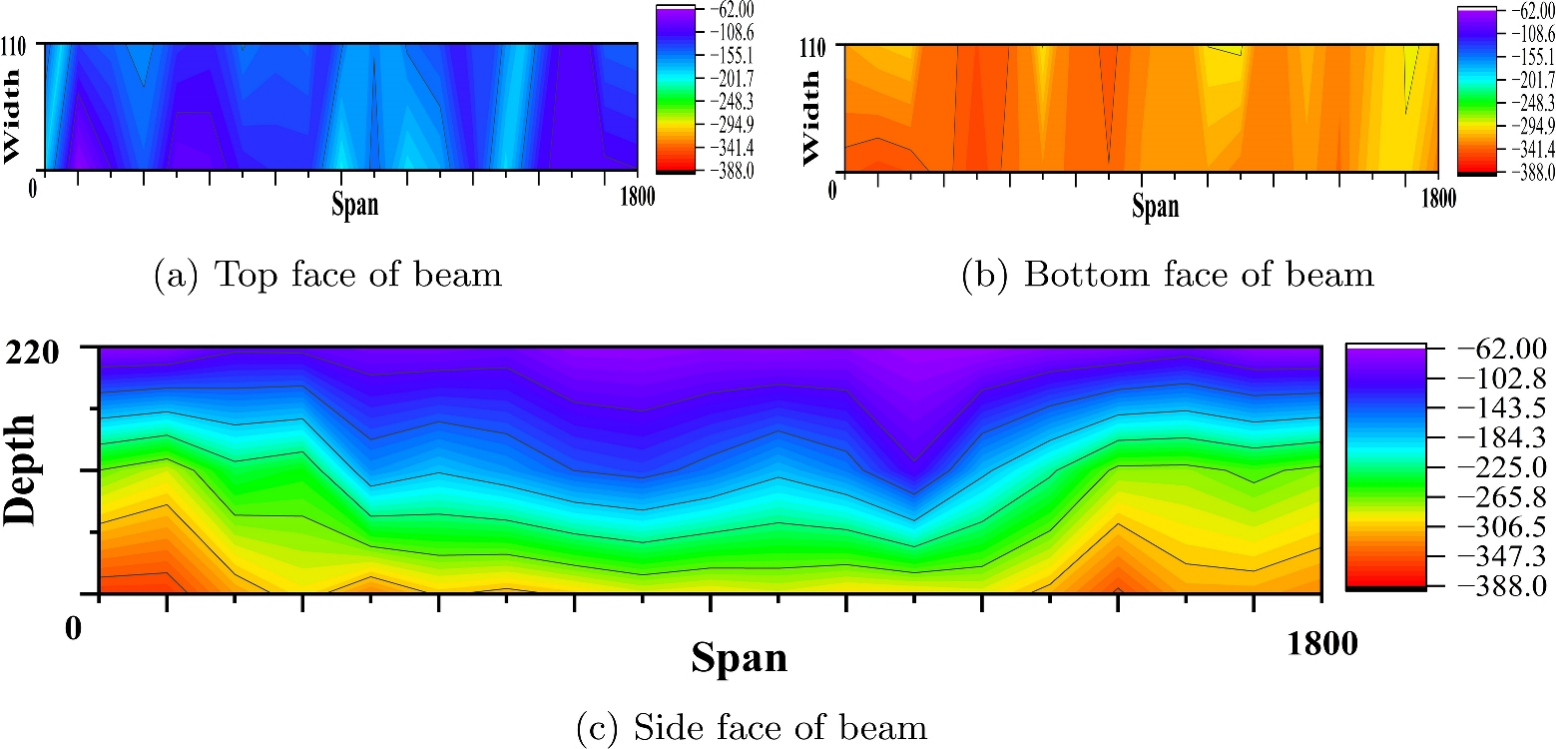
Equipotential plot of corroded PSC beam made of M60 SCC with 0.15% PPF (Beam dimension is in mm and potential values in mV).

#### Corrosion study by profometer-corrosion

After measuring the potentials on the concrete beam surface along the length considering all the grid points, the equipotential line is drawn. Figures 9, 10, 11, 12, 13 and 14 shows the average equipotential line for the specimens under the type of samples considered. The potential range of −600 to −400 mV represents the moist, chloride contaminated concrete. The probability of steel corrosion increases with the low potential. As per ASTM C 876-09^52^, the concrete surface areas with more negative potential than −350 mV have a 90% probability of corrosion. And 90% probability of no corrosion to the areas with positive than −200 mV. And these are the two extreme values considered for analysing the beam specimens.

Figures 9 and 12 show the equipotential line at the bottom, top and side face of the reference beam specimen of M40 and M60 SCC. The potential observed is greater than −200 mV. Figures 10 and 11 represents the contour map of corroded specimen without and with PPF of M40 SCC, it is seen that the high negative potential areas are more in the corroded beam without PPF than with PPF. Also, a high negative potential value observed in the beam without PPF. Figures 13 and 14 present a contour map of the corroded specimen without and with PPF of M60 SCC. In these results also it is observed that a corroded beams with fibre showed less negative potential compared to corroded beams with fibre. The presence of fibre increased the resistance of the PSC beam against the capillary action of the electrolyte correspondingly reducing the chloride contamination height, which in turn reduced the corrosion possibility.

**Table 6:** The result of bending test on PSC beam specimens.

SI. no.	Specimen type	Specimen	Cracking		Ultimate	
		Number	Load (kN)		Load (kN)	
			Load	Average	Load	Average
1	Reference PSC beam of M40 SCC	1	39.5	40.8	113	113.8
		2	42.1		114.76	
2	Corroded PSC beam of M40 SCC	1	32	32.65	102	103.39
		2	33.3		104.78	
3	Corroded PSC beam with fibre of M40 SCC	1	44.18	42.84	107.52	106.76
		2	41.5		106	
4	Reference PSC beam of M60 SCC	1	54.06	53.03	135.22	134.61
		2	52		134	
5	Corroded PSC beam of M60 SCC	1	52.98	50.99	129.7	129.1
		2	49		128.5	
6	Corroded PSC beam with fibre of M60 SCC	1	51	52.1	132	132.5
		2	53.2		133	

**Fig. 15:**
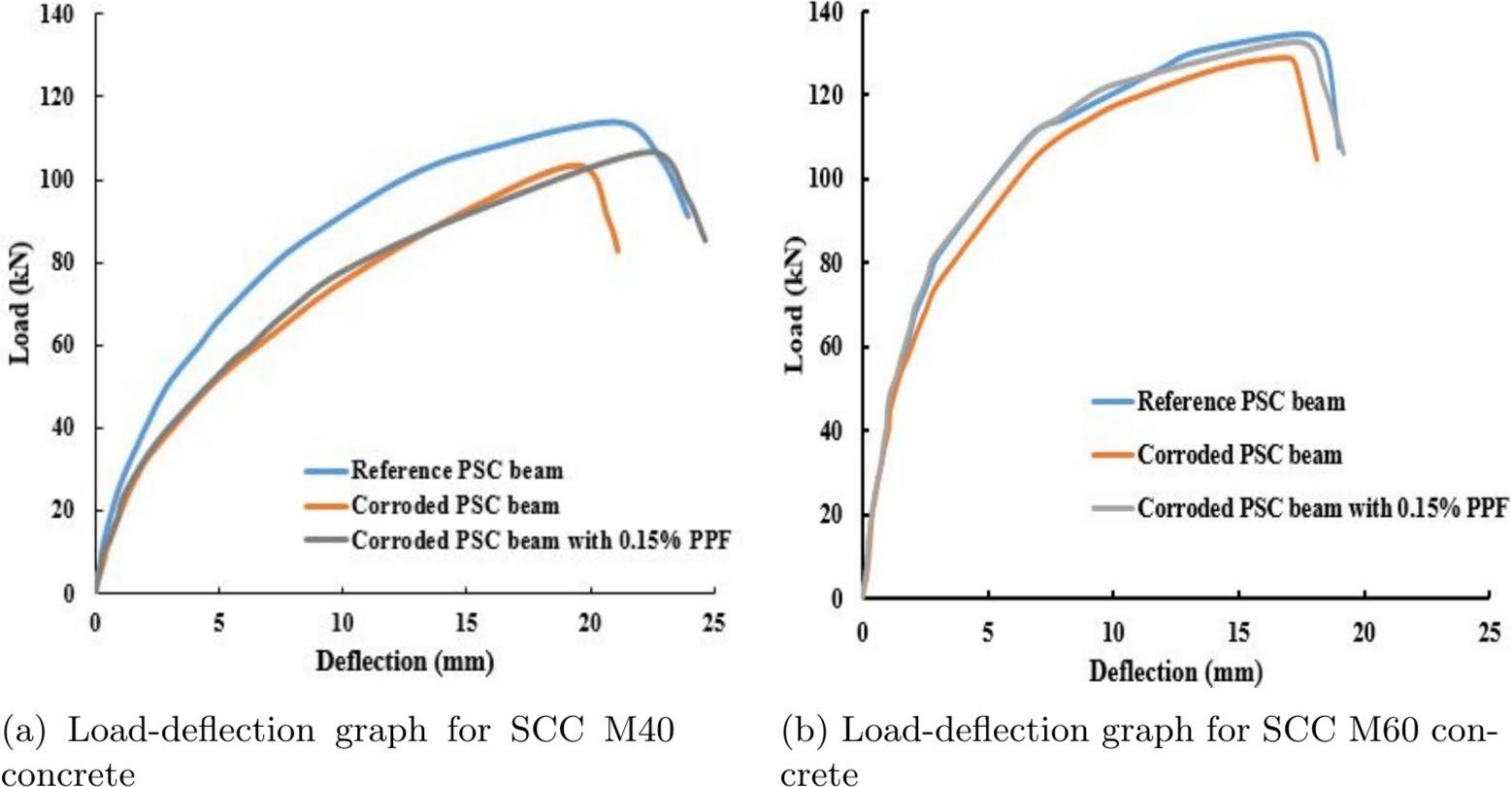
Load-deflection graph for PSC beam specimens.

**Fig. 16:**
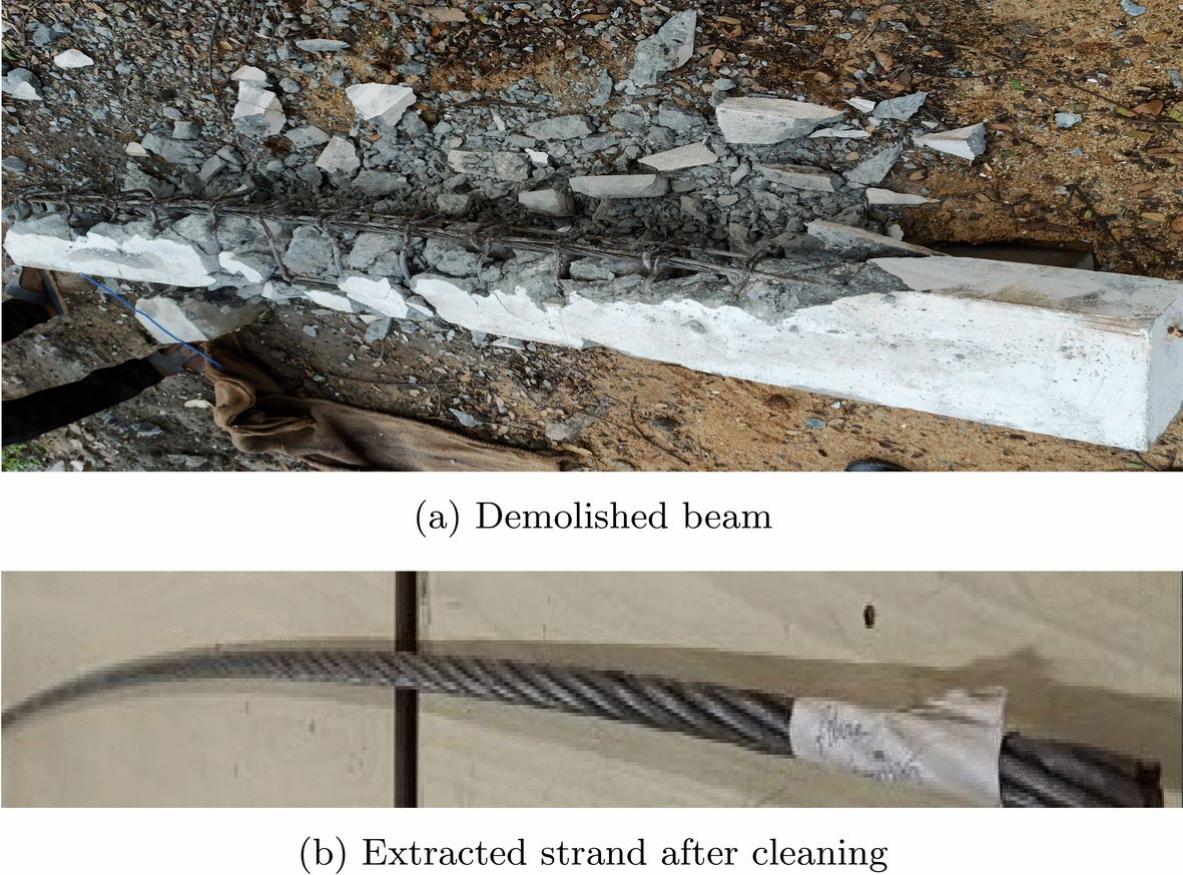
Extraction of strand by demolishing the beam for mass loss study.

**Table 7:** The experimental results of M40 SCC PSC beam specimens.

SI. no.	Parameters	Reference PSC beams	Corroded PSC beams	Corroded PSC beam with 1.5% PPF
1	Corrosion	0	3.8%	2.9%
2	Cracking load	40.801 kN	32.65 kN	42.84 kN
3	Ultimate load	113.88 kN	103.39 kN	106.76 kN
4	Ultimate deflection	21 mm	19.5 mm	22.3 mm
5	Number of cracks at beam bottom face	10 nos.	9 nos.	11 nos.
6	Maximum crack width at beam bottom face	2.5 mm	3 mm	1.5 mm

**Table 8:** The experimental results of M60 SCC PSC beam specimens.

SI. no.	Parameters	Reference PSC beams	Corroded PSC beams	Corroded PSC beam with 1.5% PPF
1	Corrosion	0	0.8%	0.5%
2	Cracking load	53.03 kN	50.99 kN	52.1 kN
3	Ultimate load	134.61 kN	129.1 kN	132.5 kN
4	Ultimate deflection	17.58 mm	16.9 mm	17.4 mm
5	Number of cracks at beam bottom face	8 nos.	7 nos.	9 nos.
6	Maximum crack width at beam bottom face	3.5 mm	4 mm	1.5 mm

### Flexural strength test results

The results obtained from the four point bending test are tabulated in the Table 6. The results show that strand corrosion in the PSC beam reduces the cracking load and ultimate load of the beam. It is also observed that the adding of fibres to the beam increased the cracking load even with the strand corrosion. This could be because of the bridging effect of fibre. When microcracks develop in the concrete by loading effect, the fibres in the vicinity will delay the propagation of cracks to the surface by arresting the microcracks by bridging effect.

The average values of load-deflection for the considered specimens(reference, corroded beam and corroded beam with fibre) are plotted in Fig. 15a and b for concrete mix M40 and M60 SCC, respectively. It is observed that the ultimate load and deflection of the corroded PSC beam are reduced by 9.21% and 7.14% respectively, for the corrosion level of 3.8%. The ultimate load reduced by 2.9% and the ultimate deflection increased by 6.19% respectively, for the corrosion level of 2.9% for the corroded PSC beam with fibre when compared with reference PSC beam specimen made of M40 SCC. The ultimate load and deflection of the corroded PSC beam is reduced by 4.09% and 3.86% respectively for the corrosion level of 0.8%. The ultimate load and ultimate deflection reduced by 1.56% and 1.02% respectively for the corrosion level of 0.5% for the corroded PSC beam with fibre, when compared with reference PSC beam specimen made of M60 SCC. The corroded PSC beam with fibre has not had much reduction in ultimate load and ultimate deflection due to PPF. When compared to the failure of the beam specimen observed, the corroded PSC beam showed abrupt brittle failure than the reference PSC beam. But the beam with fibre slightly retained the ductile failure. The PSC beams made of M60 concrete showed slightly less deflection with a higher ultimate load than the PSC beam made of M40 SCC mix. This is due to less water cement ratio and high strength in M60 SCC concrete, which makes the concrete brittle. The PSC beam having corroded strand less than 7% shows ductility reduction and experience failure abruptly due to damage of concrete and strand^6^. The increase in the corrosion level changes beam failure from ductile to brittle failure.

### Quantification of corrosion level in strand

The corrosion level in the strand is quantified after the flexural test on the PSC beam specimens. After the bending test, the strand is extracted from the beam by demolishing the beam. Figure 16 shows the image of the destructed beam to extract the strand. After extraction, strands are cleaned properly and the final weight of the strand is measured, and then mass loss is calculated. The experimental average mass loss obtained for the specimens made of M40 SCC is 3.8% for corroded PSC beam without fibres and 2.9% for corroded PSC beam 0.15% PPF. The mass loss of 0.8% for corroded PSC beam without fibres and 0.5% for corroded PSC beam with 0.15% PPF for the specimens made of M60 SCC. However theoretical mass loss considered is 6.5% for a constant current of 0.55 A and a fixed duration of 193 hrs, as per Faraday’s equation. The average corrosion level and the results obtained from the bending test are tabulated in Tables 7 and 8.

The PSC beam with fibre showed less corrosion level than the PSC beam without fibre for accelerated corrosion with a constant current and constant period. This is because of the bridging effect of uniformly spread PPF. The nonuniform spreading of fibres creates more voids and increases concrete contamination possibility. The PSC beam with M40 SCC experienced a comparatively more corrosion rate than the PSC beam made of M60 SCC with and without fibre. The better quality of concrete can resist the ingress of external agents into it.

### Energy absorption capacity of beams

**Fig. 17:**
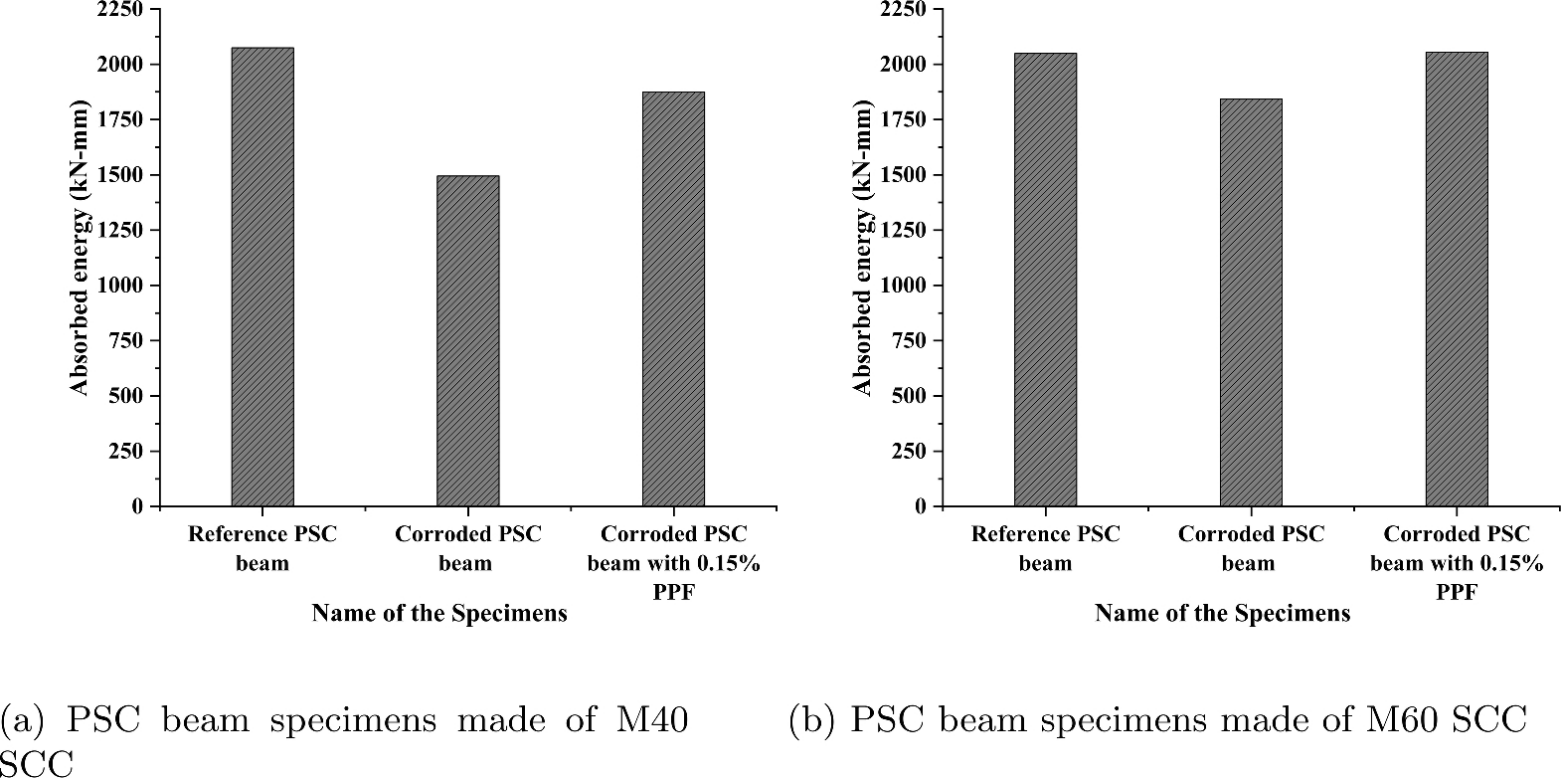
Energy absorbed by the PSC beam specimens.

The energy absorption capacity (EAC) represents the energy consumed by a unit cross sectional area of specimens at any displacement limiting point^53^. EAC for any material is considered as the area under load-deflection curve. In this study, the total area under the ascending curve and the area up to 80% of the peak load in the descending curve are considered for calculating the EAC of all the specimens. The reason is for not being able to record the readings in each stage after crossing the peak load due to the instrument limitation. So the EAC for all the specimens is calculated from the total area of curve covered from 0 to 80% of peak load value in load-deflection curve, and the values obtained are represented in Fig. 17. From Fig. 17a, it is observed that the corrosion of steel reduced the EAC of the corroded PSC beam when compared with the reference beam by 27.95% for a 3.8% corrosion level. A similar result is presented in^54^. The corroded PSC beam with 0.15% fibre with a corrosion level of 2.9% showed only a 9.67% reduction in EAC when compared with the reference PSC beam made with M40 SCC. The addition of fibres, improved the EAC of the corroded PSC beam. The EAC variation for the PSC beam made with M60 SCC is shown in Fig. 17b. The EAC for the corroded PSC beam specimen made of M60 SCC reduced to 10% for the corrosion level of 0.8%, and the corroded PSC beam with fibre showed not much difference with a corrosion level of 0.5% when compared with the reference PSC beam made of M60 SCC.

### Stiffness of beams

**Fig. 18:**
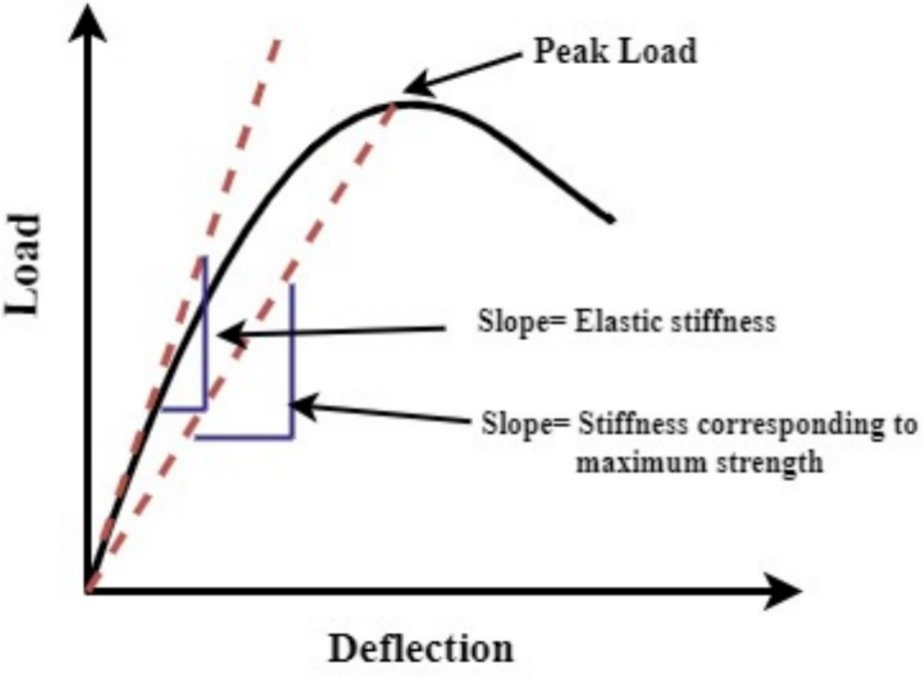
Representation of considered stiffness for study^54^.

**Fig. 19:**
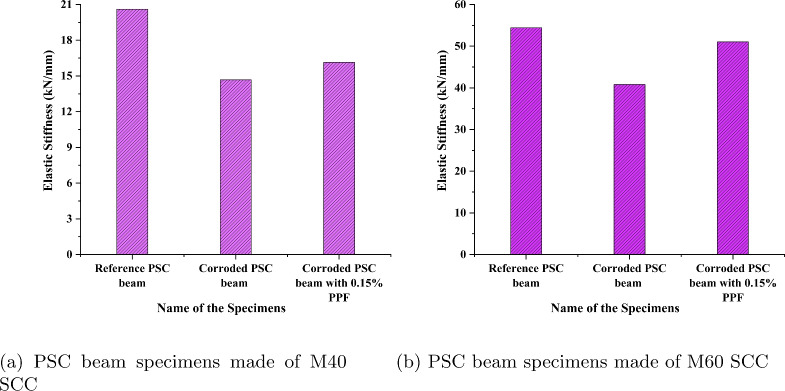
Elastic stiffness of the PSC beam specimens.

The stiffness of concrete structure represents its ability to resist deformation, it is one of the main safety factors for the serviceability of concrete. In present work, two types of stiffness are considered namely, elastic stiffness and stiffness corresponding to maximum strength to study the effect of corrosion represented in Fig. 18. The slope of the linear portion of the load-deflection curve is considered as elastic stiffness. The slope of the line passing from the origin of the load deflection curve to the load at the peak is taken as stiffness corresponding to maximum strength. Figure 19a and b presents the variation of elastic stiffness for the considered specimens. An elastic stiffness reduction of 28.8% and 25% is observed in the corroded PSC beam specimens made of M40 SCC and M60 SCC respectively, when compared with the corresponding reference beam specimens. The corroded PSC beam specimens with fibre retained slightly more elastic stiffness than the corroded PSC beam specimens. The strand corrosion has significantly less effect on the elastic stiffness. The stiffness at the elastic region is more affected by the concrete properties, so the compressive strength and fibre addition affect the variations of elastic stiffness. However, the strand corrosion of steel may not affect the elastic stiffness but reduction in the strength of concrete due to deterioration will affect it.

**Fig. 20:**
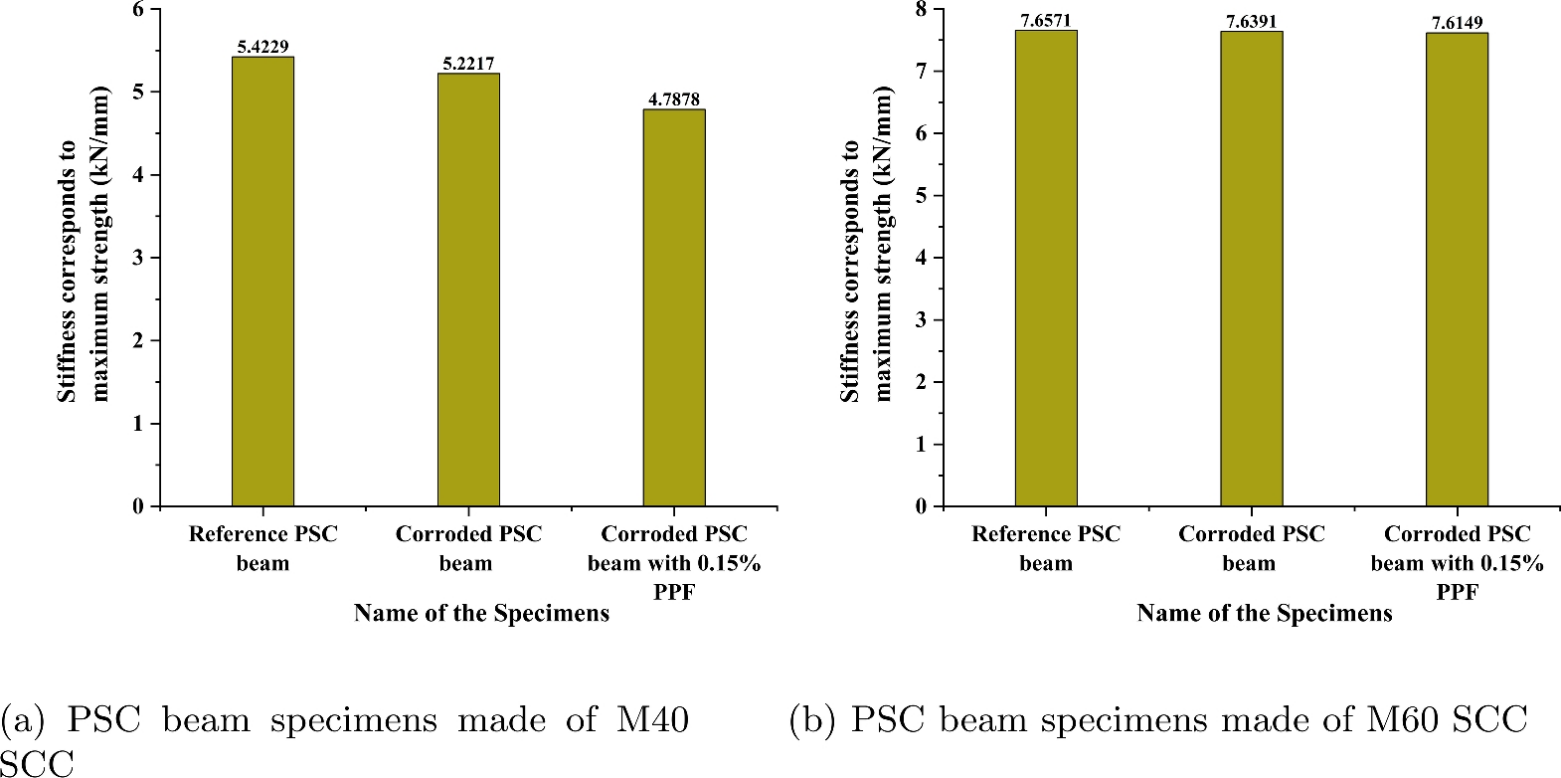
Stiffness correspond to maximum strength of the PSC beam specimens.

Figure 20 represents the variation of stiffness corresponding to maximum strength for considered PSC beam specimens. The considerable reduction in the stiffness corresponding to maximum strength is observed for specimens made of M40 SCC shown in Fig. 20a. The stiffness reduced by 3.71% for specimens corroded without fibre and 11.71% for the specimens corroded with fibre when compared with the reference PSC beam. Figure 20b presents the values for PSC beam made of M60 SCC. The stiffness reduced by 0.235% for specimens corroded without fibre and 0.55% for the specimens corroded with fibre when compared with the reference PSC beam made of M60 SCC. The stiffness corresponding to maximum strength takes into consideration the nonlinear part of the load deflection curve. After the elastic region, the load is resisted by the steels present in the beam. Thus, reduction in the cross section of the strand by corrosion makes it less stiff corresponding to the maximum strength. Here the PSC beam specimen made of M40 SCC underwent more corrosion, so it showed slightly more variation in stiffness. In PSC beam made of M60 showed less variation since corrosion levels are less.

**Fig. 21:**
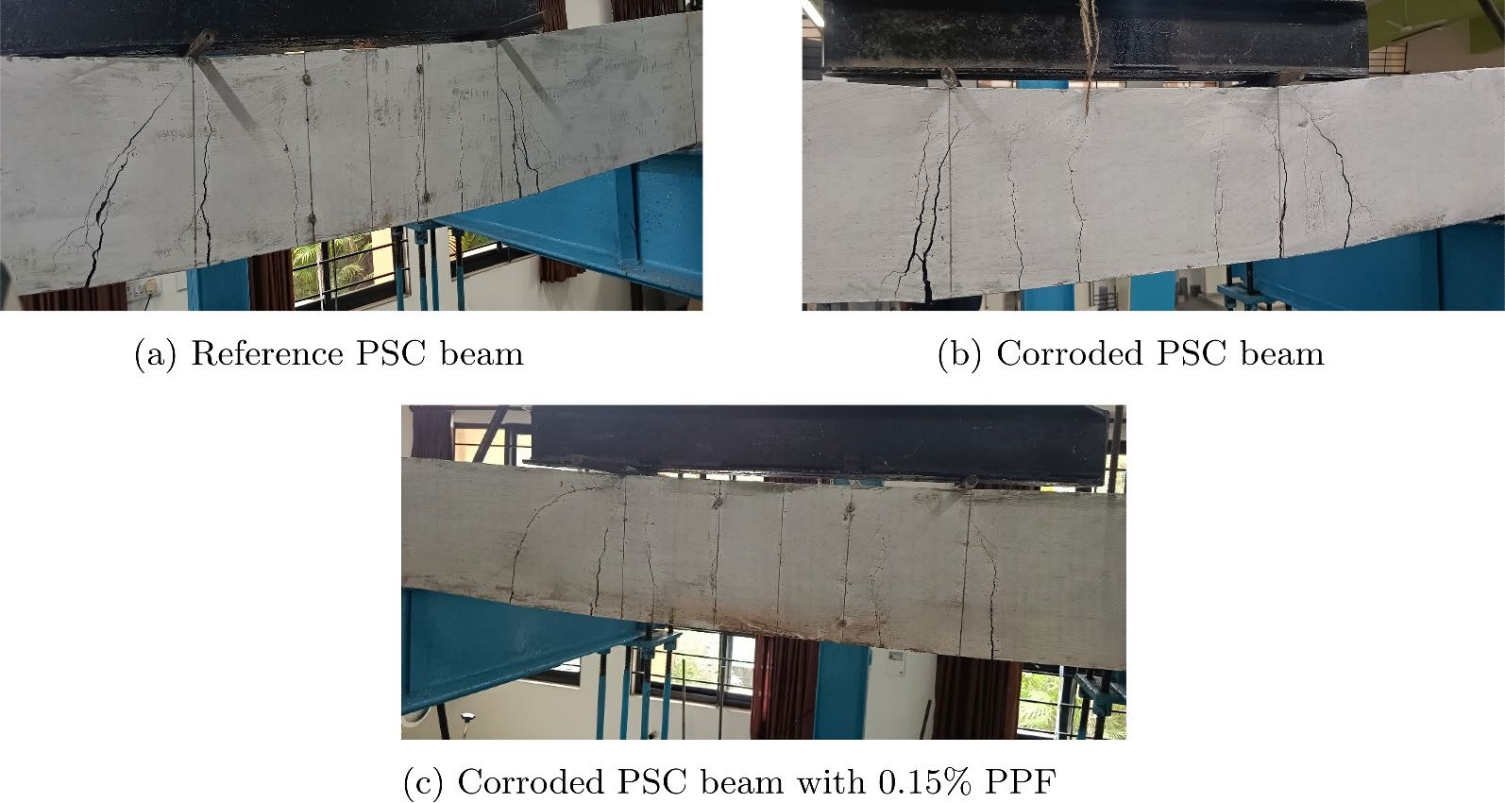
Crack patterns of PSC beam made of M40 SCC.

**Fig. 22:**
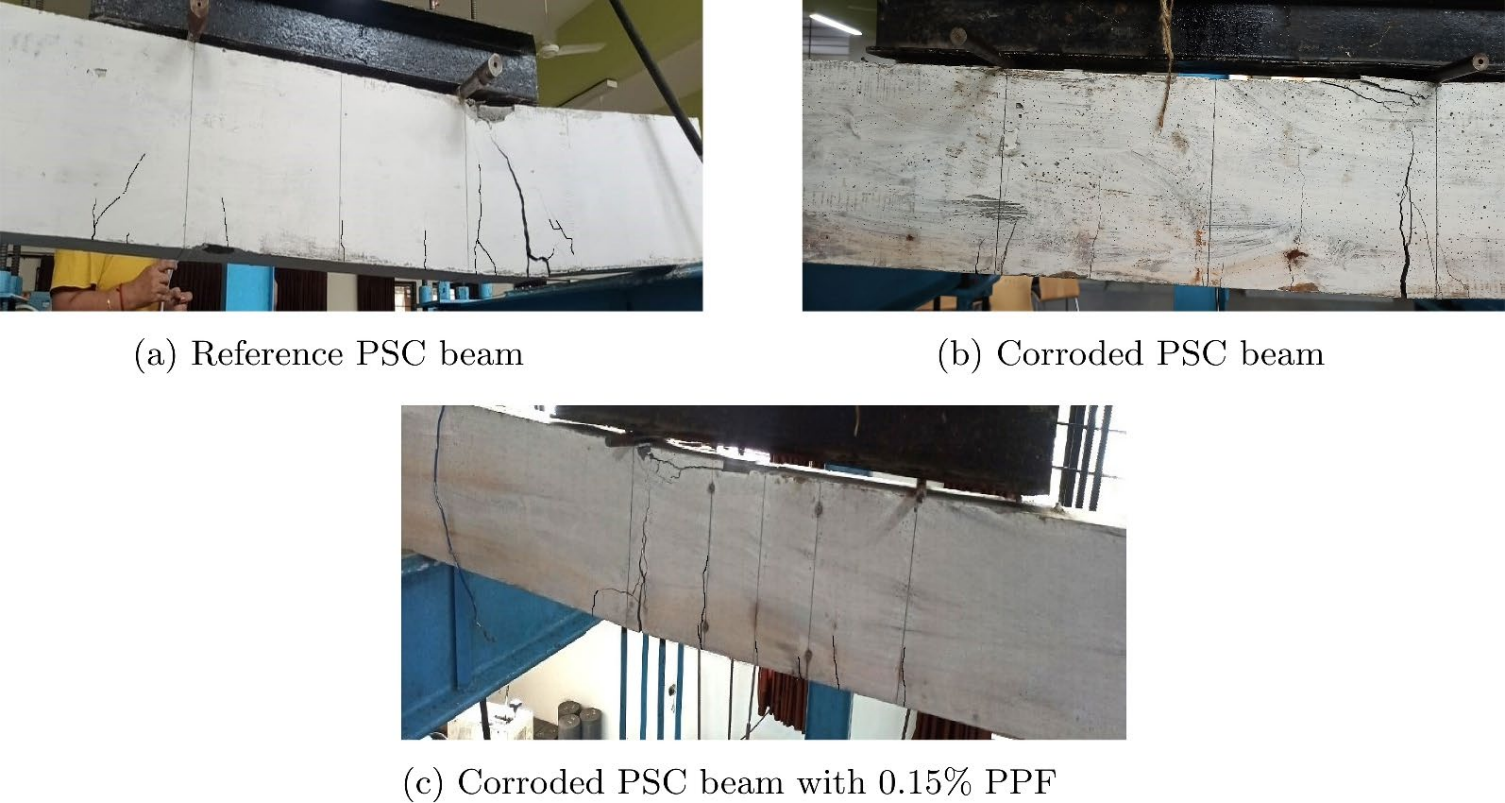
Crack patterns of PSC beam made of M60 SCC.

### Cracking pattern

The crack pattern of PSC beam specimens made of M40 SCC and M60 SCC mix under peak load is shown in Figs. 21 and 22, respectively. Flexural cracks and flexural-shear cracks are mostly seen in all the PSC beam specimens and specimens failed by concrete crushing. Also, it is observed that the number of cracks reduced in the M60 SCC specimens compared to the M40 SCC specimens. Reduction in the water cement ratio to get the high strength makes the concrete act brittle when compared with lower strength concrete. Figure 21a shows the crack patterns in the reference beam made of M40 SCC, which had no corrosion in the strand. First crack which is the flexural crack occured at an average load of 40.801 kN. Later as the load increased the cracks are obtained mainly at the middle two third of the beam span, and crack width increased with the increase in load. At the end of the peak loading stage flexural-shear cracks are obtained. At the time of peak loading the average crack width at the bottom face is found to be 2.5 mm. Figure 21b is the image of corroded beam without fibres with 3.8% corrosion level, the average load for first crack is obtained as 32.65 kN. The maximum crack width measured at the peak load stage is 3 mm. More brittle failure with less crack numbers are observed in corroded PSC beam specimens compared to reference PSC beam. The crack pattern obtained in corroded PSC beam with fibre with corrosion level 2.9% is shown in the Fig. 21c. A cracking load of 42.84 kN and crack width of 1.5 mm after the peak loading stage is obtained. The presence of fibre in concrete increased the cracking load and reduced the cracking width, less brittle failure is obtained when compared with the reference beam.

Figure 22a shows the crack pattern of the reference PSC beam made of M60 SCC. The average cracking load for this type of specimen obtained is 53.03 kN, and the average width of the crack after the ultimate load is measured as 3.5 mm. In the middle 2/3rd span of the beam flexural and flexural-shear cracks occurred at the stage of peak loading, and later stage flexural-shear cracks got widened and concrete crushed under load at the right side and more brittle failure of beam occurred without much widening of flexure cracks. A similar pattern occurred in the corroded PSC beam of M60 SCC mix without fibre with a corrosion level of 0.8% as shown in Fig. 22b. The average cracking load of 50.99 kN is obtained, and at the peak load stage the average maximum crack width of 4 mm is observed. Figure 22c presents the crack image of a corroded PSC beam with 0.15% fibre of mix SCC 60 with a corrosion level of 0.5%. The average crack load of 52.1 kN is obtained for this beam, and a maximum crack width of 1.5 mm is measured at the time of peak load stage. In this beam only flexural cracks are mainly seen and the beam top crushed under the left side load.

From the study, it is observed that, the corrosion of the strand reduced the cracking load, ultimate load and ultimate deflection of the PSC beam. But, the addition of PPF fibres to the concrete reduced the corrosion level of the PSC beam at a constant time for constant current. Also, addition of fibres improved the ultimate load and ultimate deflection corroded PSC beam and showed slight improvement in the failure mode of PSC beam. The presence of PPF fibres spreading uniformly throughout the section of PSC beam entraps the voids and microcracks in the beam and also increases the propagation time of cracks on the surface, in turn increased the performance of corroded PSC beam with 0.15% volume fraction of fibre over corroded PSC beam without fibre. Also, addition of fibre reduced the brittleness of M40 and M60 SCC concrete which is observed in studying the crack pattern of all the specimens.

## Conclusion

The conclusions from this study are highlighted as follows:

- The comparison study of PSC beams with and without PPF after accelerated corrosion test showed that corrosion levels obtained in the corroded PSC beam specimens with fibre are less than the corroded PSC beam specimens without fibre at a constant period with a constant current. The corrosion level has reduced from 3.8% to 2.9% in the PSC beam specimens made of M40 SCC with 0.15% volume fraction of PPF and the corrosion level has reduced from 0.8% to 0.5% in the specimens made of M60 SCC with 0.15% volume fraction of PPF.
- The corrosion levels reduced in the PSC beam specimens with higher grade concrete than the lower grade concrete. The corrosion levels obtained in M60 SCC concrete are less than the M40 SCC concrete specimens for constant period with constant current in accelerated corrosion test.
- Corrosion reduced the flexural strength of PSC beam. The ultimate strength of corroded PSC beam without fibre reduced by 9.21% for the corrosion level of 3.8% when compared with reference PSC beam for the specimens made of M40 SCC. The ultimate strength of corroded PSC beam with fibre reduced by 6.25% for the corrosion level of 2.9%.
- The ultimate strength of the corroded PSC beam without fibre was reduced by 4.09% for the corrosion level of 0.8% and ultimate strength of the corroded PSC beam with fibre was reduced by 1.56% for the corrosion level of 0.5% when compared with the reference PSC beam for the specimens made of M60 SCC.
- Corrosion of strand reduced the cracking load, ultimate load and ultimate deflection of PSC beam specimens. Also, corrosion has reduced the energy absorption capacity, stiffness of PSC beams.
- All the PSC beam specimens considered have failed due to the crushing of concrete under four point bending test and more brittle failures have seen in M60 SCC PSC beam specimens when compared with M40 SCC PSC beam specimens. Corroded samples without fibre have shown more brittle failure with less number of cracks than the reference PSC beam specimens and specimen made of corroded PSC beam with fibre.
- Addition of PP to the SCC mixes improved the corrosion resistance of PSC beam and marginally improves the flexural performance of corroded PSC beam.

## Data Availability

The data presented in this study are available on request from the corresponding author.
